# The effects of myricitrin and vitamin E against reproductive changes induced by D-galactose as an aging model in female mice: An experimental study

**DOI:** 10.18502/ijrm.v17i10.5486

**Published:** 2019-11-28

**Authors:** Mina Omidi, Akram Ahangarpour, Seyed Ali Mard, Layasadat Khorsandi

**Affiliations:** ^1^Department of Physiology, Faculty of Medicine, Student Research Committee, Ahvaz Jundishapur University of Medical Science, Ahvaz, Iran.; ^2^Physiology Research Center, Department of Physiology, Faculty of Medicine, Ahvaz Jundishapur University of Medical Sciences, Ahvaz, Iran.; ^3^Department of Anatomical Sciences, Faculty of Medicine, Cellular and Molecular Research Center, Ahvaz Jundishapur University of Medical Sciences, Ahvaz, Iran.

**Keywords:** Aging, D-galactose, Mice, Myricitrin, Vitamin E.

## Abstract

**Background:**

Aging is accompanied by decreasing general function in the cells and tissues. D-galactose (D-gal) induces aging and plays a role in the pathogenesis of it. Myricitrin is a plant-derived antioxidant.

**Objective:**

The present study was performed to evaluate the effects of myricitrin on antioxidant defense, sex hormone levels, uterus, and ovarian histology in D-gal-induced aging female mouse model.

**Materials and Methods:**

In this experimental study, 72 female adult NMRI mice, weighing 30-35 gr, 3-4 months old, were randomly divided into six groups (n = 12/each): (I) Control (vehicle; normal saline), (II) D-gal at 500 mg/kg/d for 45 days, (III-V) D-gal + myricitrin-treated groups (these groups received myricitrin at 5, 10, and 20 mg/kg/d, and (VI) D-gal + 100 mg/kg/d vitamin E orally for the last 28 days. The antioxidant indices were done on the basis of colorimetric method, and sex hormone levels were measured by using enzyme-linked immunosorbent assay kits. Histological assessment of the uterus and ovaries were also evaluated.

**Results:**

D-gal impaired the estrous cycle, also degenerative changes occur in the ovarian follicles and damage to the uterus and ovarian tissue occurs. In D-gal group, the level of sex hormones (p = 0.03) and the total antioxidant capacity (p = 0.002) decreased, while the level of malondialdehyde and gonadotropins increased (p = 0.03). Myricitrin at lower doses and vitamin E ameliorated the D-gal effects.

**Conclusion:**

These findings suggest that myricitrin at low doses can effectively prevent D-gal-induced oxidation and aging in mice. The effect of myricitrin was equivalent and sometimes better than vitamin E.

## 1. Introduction

Disturbance in antioxidant systems may lead to pathological outcomes in women. An antioxidant supplement has been shown as a plan for treating reproductive and infertility diseases with oxidative stress control (1). The accelerated aging of D-galactose (D-gal) in mice has been widely used to study aging mechanisms and drug screening (2). This model has a shorter life span and aging features. Regular injection of D-gal solution into the rat can be a sign of the natural aging models used in the screening of anti-aging drugs and their drug activities (3). D-gal causes to weaken the immune system; a deficiency in sex hormones; an increment in reactive oxygen species (ROS), malondialdehyde (MDA), and declined superoxide dismutase, catalase function, and the level of inflammatory cytokines (4).

Health depends on the balance between free radicals and antioxidant defense. Aging and age-related illnesses are reflected as an inadequate antioxidant defense for the body to deal with oxidative stress during the time. Free radical/oxidative stress theory states that because of the misbalance of free radical metabolism, the structure and function of tissues and organs decrease and accelerate aging (5).

D-gal plays a role as a reducing natural sugar that reacts with amino groups in biomolecules (6, 7). Myricitrin, a flavonol glycoside, is plenty in the fruits, branches, bark, and leaves of plants like *Myrica cerifera* and Ampelopsis *Eugenia uniflora* (8). Myricitrin -due to its high antioxidative, antiviral and antibactericidal properties- is widley used in foods, cosmetics, and medicines (6). The most reported effects of myricitrin are antinociceptive, anti-inflammatory, anti-allodynia, neuroprotective and antioxidative properties (8). The protective effects of myricitrin in endothelial cells against ROS-induced apoptosis and atherosclerosis formation in ApoE/mice have been reported (9). In vitro studies have been shown effects of myricitrin including anticarcinogenic, antinociceptive, reduced oxidative stress, cytotoxicity in some tissues and cells (6), production of antioxidant, antidiabetic and antiapoptotic effects (10). Myricitrin is a phytochemical agent that regulates fertility with the ability to protect DNA from cell damaged by oxidative stress (11).

Considering the antioxidant activity of myricitrin and the effects of D-gal to induce an aging model of the animal through excessive production of ROS and an antioxidant deficiency and the effects of free radicals on the reproductive system, this experimental study investigated the effects of myricitrin on the reproductive system of female mice in D-gal aging models.

## 2. Materials and Methods

### Drugs

The reagents are as follows: Myricitrin, purity 98% (AvaChem Scientific, USA), D-gal (Merck, Germany), progesterone, 50 mg\ml, (Iran hormone, Iran), Estradiol, valerate, 10 mg\ml, (Aburaihan, Iran), and vitamin E, (Osve, Iran).

### Animals and experimental details

The experimental study on 72 female NMRI mice, weighing 30-35 gr, 3-4 months old, were obtained from the animal house of the Ahvaz Jundishapur University of Medical Sciences (AJUMS). The animals had free access to tap water and food with a 12-hr light/dark cycle and controlled temperature (22 ± 2°C) all over the experiment. The mice were randomly distributed into six groups (n = 12/each) as follows:

I. Control group: Mice received subcutaneously (s.c.) vehicle; normal saline for 45 days (0.1 ml).

II. D-gal group: Mice were injected D-gal (s.c., 500 mg/kg/d for 45 days) (4).

III. Mice were injected D-gal and myricitrin (5 mg/kg/d; by gavage for the last 28 days).

IV. Mice were injected D-gal and myricitrin (10 mg/kg/d; by gavage for the last 28 days).

V. Mice were injected D-gal and myricitrin (20 mg/kg/d; by gavage for the last 28 days).

VI. Mice were injected D-gal and vitamin E (100 mg/kg/d; by gavage for the last 28 days) (12).

### Estrous cycle

The cycle of rodent breeding is called the estrous cycle, which is short and accurate. These four stages include proestrus, estrus, metestrus, and diestrus, and last for 4 to 5 days (13). In the beginning of experiments to match the estrous cycle in the mice, each mouse received an intramuscular injection of 100 mg of estradiol valerate (dissolved in 0.2 mL olive oil). After 42 hr, 50 mg of progesterone was injected intramuscularly (4). The stages of the estrous period in each mouse were determined by examining vaginal smear. For this, vaginal sampling was performed using a wet swab with normal saline. The smears were constant on slides and stained with 1% aqueous methylene blue, after which they were evaluated microscopically.

### Analysis of tissue

The mice were weighed and anesthetized with ketamine/xylazine. After the blood samples were gathered from the heart and centrifuged, the plasma was separated and kept at -70°C for hormonal evaluations. The uterine and ovarian tissues of each mouse were removed and organ weight/body weight ratio was accounted. The segments of uterus and ovary were fixed in 10% formalin for histological studies.

### Hormonal assessment

Enzyme-linked immunosorbent assay (ELISA) kits were used for measuring levels of estrogen and progesterone (DiaMetra, Italy). Mouse Follicle-stimulating hormone (FSH) and Luteinizing hormone (LH) levels were evaluated using the ELISA assay kits (Cusabio China Inc., Wuhan, China). The sensitivity of estrogen, progesterone, FSH and LH were 10 pg/mL, 0.05 ng/mL, 2.5 and 0.5 mIU/ml respectively. The detection range FSH and LH were 4-140 and 2-75 mIU/ml, intra- and interassay coefficient of variation was < 15%.

### Antioxidant enzyme activities

The TAC concentration levels in the plasma were assessed using the ELISA assay kits (ZellBio GmbH, Germany) in accordance with the manufacturer's instructions. The TAC concentration assays can be used to determine the TAC concentration within a range of 0.125-2 mM with 0.1 mM sensitivity, intraassay CV < 3.4%, and interassay CV < 4.2%. MDA assessment in uterine and ovarian tissues was performed using ELISA assay kit (ZellBio GmbH, Germany). The assay detects the MDA level calorimetrically in a range of 0.78-50 µM with 0.1 µM sensitivity, intraassay CV 5.8%, and interassay CV7.6%.

### Histology assessments

In 10% formalin solution the mouse ovary and uterus tissues were fixed. The tissues were dehydrated in graded alcohol concentrations and embedded in paraffin. Sections of 4-6 μm were collected and stained with hematoxylin & eosin (H&E). Six microscopic H&E-stained slides per animal were assayed for the assessment of histological variations (14).

### Ethical consideration

All procedures involving animals were approved by the Animals Committee of AJUMS, and conducted in accordance with the guide for the Care and Use of Laboratory Animals (IR.AJUMS.REC.1395.639).

### Statistical analysis

The results are expressed as the mean ± standard error of the means. One-way ANOVA followed by post-hoc Tukey test (SPSS version 15, Chicago, IL, USA) was used to evaluate the differences between the different groups. A p-value < 0.05 was considered statistically significant in each group.

## 3. Results

### Effects of myricitrin on estrous cycle

D-gal impaired the estrous cycle such as aging. The administration of myricitrin at 5 mg/kg for 28 days improved the impairment of the estrus cycle in D-gal-treated mice.

### Effects of myricitrin on the histology of the ovarian and uterine tissues and weight

**Figure 1:**
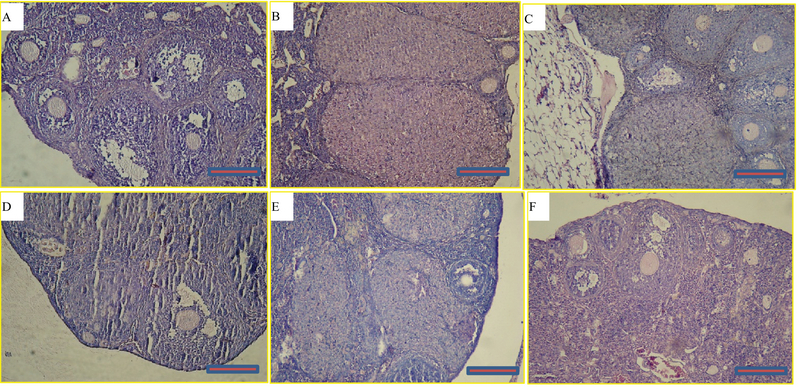
Light microscopy of cross-sections of hematoxylin- and eosin-stained ovaries from the control and experimental groups: (A) Control, (B) D-gal, (C) D-gal + 5 mg/kg myricitrin, (D) D-gal + 10 mg/kg myricitrin, (E) D-gal + 20 mg/kg myricitrin, and (F) D-gal + 100 mg/kg vitamin E. Scale bars: 100 µm.

**Figure 2:**
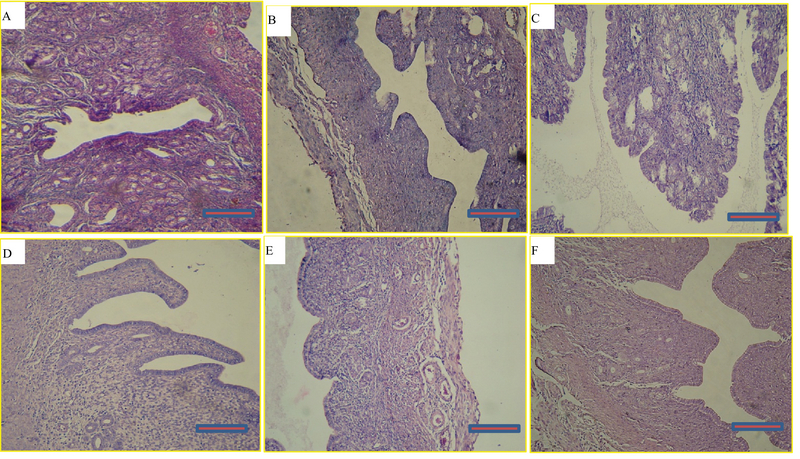
Light microscopy of cross-sections of hematoxylin- and eosin-stained uterus from the control and experimental groups: (A) control, (B) D-gal, (C) D-gal + 5 mg/kg myricitrin, (D) D-gal + 10 mg/kg myricitrin, (E) D-gal + 20 mg/kg myricitrin, and (F) D-gal + 100 mg/kg vitamin E. Scale bars: 50 µm.

A microscopic assessment by H&E demonstrated that in the control group, the ovaries included Graafian follicles with a clear zona pellucida. In the D-gal-treated mice ovaries, degenerative variations obtained in the follicles. In the D-gal group the Graafian follicle and zona pellucida were not evident. In the D-gal + 5 mg myricitrin group, Graafian follicle with an obvious zona pellucida was observed. In D-gal + 10 mg myricitrin group, the number of growing follicles seemed to be less than D-gal + 5 mg Myricitrin. In the D-gal + 20 mg myricitrin group follicular degeneration in different stages was evident. In the D-gal + 100 mg vitamin E group, growing follicles in different stages were observed (Figure 1). The uterus of the control mice had normal histological architecture. Atrophy of the endometrium and decrease in the glands number was observed in the D-gal group. In the D-gal + 5 mg myricitrin group, the number of glands and the height of endometrium seemed to be more than the D-gal-treated mice. The appearance of the uterus in both the D-gal + 10 mg myricitrin and the D-gal + 20 mg myricitrin groups was similar to the D-gal-treated mice. The architecture of the uterus in the D-gal + 100 mg vitamin E groups was similar to the D-gal + 5 mg myricitrin group (Figure 2). The ovary and uterus weight/body weight percentage did not change significantly in the intra-group comparison (Table I).

### Effects of myricitrin on the plasma levels of sex hormones

The plasma level of estrogen in the D-gal-treated mice was significantly lower than in the control group (p = 0.03). Myricitrin significantly increased the estrogen level in the D-gal + 5 mg (p = 0.02), 10, and 20 mg myricitrin (p < 0.001) compared to the D-gal group. The effect of D-gal + 20 mg myricitrin on the estrogen level was similar to the effect of D-gal + 100 mg vitamin E (Table II).

D-gal decreased the progesterone level compared to the control group (p = 0.03). This level was significantly increased in the D-gal + 5 (p = 0.02), 10 (p = 0.01), and 20 mg (p < 0.001) myricitrin and Vitamin E groups (p = 0.003), compared to the D-gal group (Table II).

The plasma level of LH was significantly increased (p = 0.03) by treating the D-gal group compared with the control group. Myricitrin 5 and 10 mg (p = 0.002) decreased this level significantly in the D-gal-treated mice. The plasma level of FSH was significantly increased (p = 0.03) by treating the D-gal group compared with the control group. Myricitrin 5 mg and Vitamin E significantly (p = 0.04) decreased this level in the D-gal-treated mice. The plasma level of LH and FSH in D-gal-treated mice received the highest dose of myricitrin and were approximately equal to the D-gal group (Table II).

### Effects of myricitrin on the antioxidant indices of ovarian and uterine tissues

**Figure 3:**
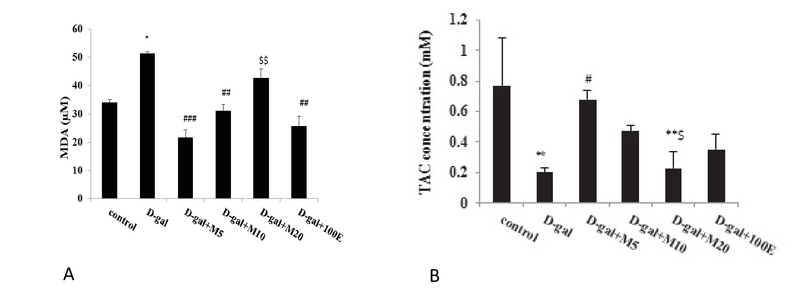
Effect of myricitrin and vitamin E on (A) plasma MDA and (B) TAC level in D-gal-treated mice. Results are presented as mean ± SEM; n = 12;${}^{*}$p < 0.05 compared with the control group. ${}^{\#\#}$p < 0.01 and ${}^{\#\#\#}$p < 0.001 compared with the D-gal group, ${}^{\$\$}$p < 0.01 compared between the treated groups.

The plasma levels of MDA in the D-gal group were significantly increased (p = 0.03) compared to the control mice (Figure 3A), while TAC activity in the D-gal group significantly decreased (p = 0.002) compared to the control group (Figure 3B). Myricitrin significantly decreased the tissue level of MDA and increased TAC. Myricitrin at 5 mg/kg significantly decreased the MDA levels (p < 0.001) and increased TAC (p = 0.04) in the D-gal mice compared to the other doses of myricitrin and vitamin E.

**Table 1:** The effect of different doses of myricitrin and vitamin E on the ovary and uterus weight/body weight percentage in the D-gal-treated mice (n = 12)


**Groups**	**Ovary and uterus/body weight (%)**
Control	0.98 ± 0.12
D-gal	0.89 ± 0.07
D-gal + M5	0.82 ± 0.06
D-gal + M10	0.86 ± 0.05
D-gal + M20	0.96 ± 0.13
D-gal + E100	0.92 ± 0.03
Mean ± SE, one-way ANOVA, and post-hoc LSD tests	
D-gal: D-galactose; D-gal + M: D-galactose+ Myricitrin; D-gal + E: D-galactose+ vitamin E	

**Table 2:** The effect of different doses of myricitrin and vitamin E on sex hormones in aging mice induced by D-gal (n = 12)


**Groups**	**LH (mlU/ml)**	**FSH (mlU/ml))**	**Estrogen (pg/ml)**	**Progesterone (ng/ml)**
Control	0.71 ± 0.18	3.38 ± 0.99	485.25 ± 121.58	18.98 ± 0.85
D-gal	1.64 ± 0.12*	6.61 ± 0.67*	131.91 ± 64.39${}^{*\$\$\$}$	17.40 ± 0.66*
D-gal + M5	0.67 ± 0.18${}^{\#\#\$\$}$	5.20 ± 0.76${}^{\#}$	209.62 ± 85.06${}^{\#\$\$}$	20.97 ± 1.08${}^{\#}$
D-gal + M10	0.82 ± 0.04${}^{\#\#\$\$}$	6.03 ± 0.57${}^{**}$	529.62 ± 119.08${}^{\#\#\#}$	21.27 ± 2.61${}^{\#\#}$
D-gal + M20	1.60 ± 0.20**	5.59 ± 0.65${}^{*}$	1049.05 ± 240.23${}^{\#\#\#}$	36.61 ± 3.14${}^{\#\#\#}$
D-gal + E100	1.49 ± 0.14${}^{**\#\#\$\$}$	4.30 ± 0.40${}^{\#}$	1016.50 ± 208.62${}^{\#\#\#**}$	25.33 ± 3.29${}^{\#\#}$
Mean ± SE, one-way ANOVA, and post-hoc LSD tests			
*P < 0.05 and **p < 0.01 vs Control; ${}^{\#}$P < 0.05, ${}^{\#\#}$p < 0.01, and ${}^{\#\#\#}$p < 0.001 vs D-gal; ${}^{\$}$P < 0.05, ${}^{\$\$}$p < 0.01, and ${}^{\$\$\$}$p < 0.001 vs D-gal + M20
D-gal: D-galactose; D-gal + M: D-galactose+ Myricitrin; D-gal + E: D-galactose+ vitamin E; LH: Luteinizing hormone; FSH: Follicle stimulating hormone			

## 4. Discussion

This study revealed that myricitrin effectively reduced the effects of aging caused by D-gal in the female mice reproductive system. The D-gal-induced aging model has been generally used for aging research because it speeds up the aging process that is similar to natural aging processes (3). D-gal has been used to generate a natural-like aging process and oxidative stress (15).

Previous studies have shown that D-gal can raise the LH and FSH levels in male mice (16). A previous study reported that D-gal can decrease estrogen and progesterone levels (4). Consistent with the previous report, the present study also indicated that estrogen and progesterone levels decreased and the levels of gonadotropins increased in the D-gal group. The present study demonstrates that D-gal could increase LH and FSH levels. The previous study revealed that D-gal can increase LH and FSH levels in male mice similar to the hormonal changes in aging (16). Myricitrin at lower doses decreased this level in D-gal-treated mice. The plasma level of LH and FSH in D-gal-treated mice received a high dose of myricitrin approximately equal to the D-gal group. Therefore, these findings show that myricitrin at 20 mg/kg was not able to treat or alleviate the impairment effects of D-gal on gonadotropins. Myricitrin at this dose could also not improve the total antioxidant capacity, while at low doses, the total antioxidant capacity can be increased. Most studies confirm the role of oxidative stress in the development of reproductive system diseases (16-18). Oxidative stress, the imbalance between the production of ROS and antioxidant defense, leads to various reproductive diseases such as endometriosis and polycystic ovarian syndrome (18).

Various antioxidant protective systems, including vitamin E, CAT and glutathione, maintain the reproductive system from ROS at physiological levels. ROS at the pathologic level disrupts the antioxidant defense and causes damage to the reproductive system (17), while at the physiological level, it plays an important role in the natural reproduction process (19). Vitamin E was initially found to be essential for reproduction, which is found in a number of foods and herbs (20). In this study, it was revealed that vitamin E protected the function of ovaries against D-gal. In agreement with this study, previous researches have shown that vitamin E acts as a strong natural antioxidant, especially in the reproductive system (21). Also vitamin E deficiency has an adverse effect on fertility and pregnancy (22). Vitamin E as a potent antioxidant improves reproductive disorders (23).

In this study, myricitrin has been shown to increase estrogen concentration and effectively reduce the effects of aging caused by D-gal. High doses of myricitrin increased estrogen and progesterone level, but gonadotropins could not be reduced, hence, the hypothalamic-pituitary-gonadal axis did not function properly. In other words, the present findings indicate that a high dose of myricitrin exerts effects on the hypothalamic and/or the pituitary gland and cannot compensate for the effect of D-gal on gonadotropin hormones. Therefore, it is probable that myricitrin cannot be useful at high concentrations for the reproductive system.

In this study, D-gal has been responsible for lipid peroxidation and thereby for increasing the content of MDA, which is a chief sign of lipid peroxidation in aging textures. On the contrary, D-gal reduces the level of antioxidants, such as TAC, causing oxidative damage. Similar to these results, D-gal accelerates the aging process by decreasing TAC and increasing MDA levels in the ovary (4). As shown in the present results, myricitrin decreased the MDA content while increasing the TAC activity. Also, in another study, showed that, myricitrin can reduce MDA contents (24). Then, D-gal causes oxidative stress, which is consistent with Yan and colleague's study that showed that D-gal reduces the number of follicles and oocytes, resulting in aging of the ovary (25).

Kassem and co-workers for the first time showed that natural myricitrin extracts protected cells and DNA structure against oxidative stress by downregulating the gene expression of cytochrome P19 and LH as well as by upregulating the gene expression of glutathione S-transferase (11). In the previous study, it has been shown that myricitrin prevented oxidative stress and cell apoptosis caused by hyperglycemia (24).

The present study also indicated that the ovary and uterus weight/body weight percentage did not change in the intragroup comparison. Perhaps the uterus and ovary have grown in proportion to the trend of weight gain (26). As the present results implied, the inability of myricitrin at dose 20 mg/kg against the deleterious effect of D-gal on ovaries and uterus functions is due to a lack of antioxidant activity at this dose. Therefore, these findings strongly reveal that myricitrin through increasing the antioxidant potency protected the ovaries and uterus against D-gal in mice.

## 5. Conclusion

This research showed that D-gal by inducing oxidative stress creates a model of aging similar to the normal female reproductive system and reduces fertility. Our results showed that myricitrin in low doses can delay the aging process by increasing secretion of estrogen and progesterone levels, reducing LH, FSH, MDA content and increasing TAC. Myricitrin also had a positive effect on the tissues of the uterus and ovaries. Therefore, myricitrin with the inhibitory effect of oxidative stress in the aging model induced by D-gal, and the suppression of free radicals and ROS, improved the age-related changes in the reproductive system. The minimal studied dose of myricitrin had the highest level of protection compared to other doses. Myricitrin at 20 mg/kg was not toxic but failed to increase the antioxidant potency. These findings suggest that myricitrin can be a candidate to alleviate the age-related changes in women.

##  Conflict of Interest

The authors have no conflicts of interest to report with respect to this paper.
